# Morpho-Physiological Responses and Secondary Metabolites Modulation by Preharvest Factors of Three Hydroponically Grown Genovese Basil Cultivars

**DOI:** 10.3389/fpls.2021.671026

**Published:** 2021-04-26

**Authors:** Michele Ciriello, Luigi Formisano, Christophe El-Nakhel, Giandomenico Corrado, Antonio Pannico, Stefania De Pascale, Youssef Rouphael

**Affiliations:** Department of Agricultural Sciences, University of Naples Federico II, Naples, Italy

**Keywords:** *Ocimum basilicum* L., floating raft system, cut, specialized metabolites, phenolic acids, volatile compounds

## Abstract

Sweet basil (*Ocimum basilicum* L.) is an economically important leafy vegetable especially in Mediterranean countries. In Italian gastronomy, the large elliptical leaves of the Genovese type are mostly used for the well-known pesto sauce, and almost all (>90%) professional production is for the food industry. The growing demand for fresh leaves with standardized technological and sensory characteristics has prompted basil producers to adopt advanced cultivation methods such as the floating raft system (FRS). The aim of this study was to evaluate the productive, qualitative, and physiological performance of three Genovese basil cultivars (“Aroma 2,” “Eleonora,” and “Italiano Classico”) in two successive harvests and at two densities (159 and 317 plants m^–2^). Caffeic, chicoric, rosmarinic, and ferulic acid were determined through the high-performance liquid chromatography (HPLC) system, whereas the extraction and quantification of the volatile organic compounds (VOCs) were performed by solid-phase microextraction (SPME) and gas chromatography coupled to a mass spectrometer (GC/MS). “Aroma 2” showed the highest fresh yield and photosynthetic rate together with the lowest nitrate content. For all the tested cultivars, the higher density, while reducing the number of leaves per plant, resulted in higher fresh and dry production per unit area, without altering the aroma profile. Successive harvests resulted in a significant increase in both the yield (37.5%) and the total phenolic acids (75.1%) and favored Eucalyptol and 1-octen-3-ol accumulation (+25.9 and +15.1%, respectively). The here presented comprehensive and multifactorial assessment of the productive and qualitative response of basil provides evidence of the positive effects (from biomass to specialized metabolites) that can be obtained from the management of the pre-harvest factors in soilless cultivation. In addition, it also highlights the role and constraints of the genetic factor in the observed response. We also discuss the implications of our work considering the impact for the food processing industry. Future research may explore the phenolic acids accumulation as a possible fortification means to extend the pesto sauce shelf life, reducing the need of added antioxidants and thermal processing.

## Introduction

Sweet basil (*Ocimum basilicum* L.) is an annual herbaceous species of the Lamiaceae family considered among the most popular Mediterranean aromatic and edible herbs (Shahrajabian et al., 2020). The genetic and morphological variability of the *Ocimum* genus has led to the classification of over 60 species (Filip, 2017), which differ in growth habits, leaf morphology, pigmentation, and aromatic content (Makri and Kintzios, 2008). Furthermore, the recent intense plant breeding has made taxonomic classification more challenging by fixing morphological natural variation in a number of different horticultural types (Dudai and Belanger, 2016). Basil has also historically been used in folk medicine as a soothing agent for stomach and intestinal discomforts. Nowadays, *O. basilicum* is used for its distinctive aroma in the food processing, cosmetic, and pharmaceutical industries (Barátová et al., 2015). In Italian cuisine, freshly picked leaves are a popular food garnish (e.g., the real pizza Margherita, Caprese salad). Specifically, the “Basilico Genovese,” which has obtained the European Union (EU) Protected Designation of Origin label (EU Reg. 611/2010), is the central ingredient of the famous green sauce worldwide known as “pesto” (Salvadeo et al., 2007; Kiferle et al., 2011). Over the last decades, the total area used for the cultivation of Genovese basil in Italy has increased by over 66%, with a 25% increase in the protected environment (Italian National Institute of Statistics (ISTAT), 2019), driven mainly by the growing demand of the food industry (Morano et al., 2017).

In aromatic plants, composition of the essential oil is a relevant qualitative feature, which can influence consumer choice (Dudai and Belanger, 2016). In sweet basil, most of the aromatic molecules are stored in trichomes and belong to (mono-)terpenes and phenylpropanoids (Marotti et al., 1996). Among the latter, linalool and methyl chavicol characterize the fine aroma of this herb (Makri and Kintzios, 2008; Bekhradi et al., 2015; Filip, 2017). Nowadays, consumer’s choice is increasingly oriented toward high-quality foods with nutritional properties (Sgherri et al., 2010; Morano et al., 2017). Recently, there has been a strong interest in the biochemical characterization of minor species that could represent a relevant source of antioxidants beneficial to human health (Ahmed et al., 2019). Basil’s high antioxidant capacity is mainly attributable to rosmarinic acid, a characteristic metabolite of several medicinal plants along with other phenolic acids (e.g., caffeic, chicoric, and ferulic acids) (Petersen and Simmonds, 2003; Makri and Kintzios, 2008; Lee and Scagel, 2009; Salachas et al., 2015). The phenolic composition and the aromatic bouquet of basil are also strongly affected by the genetic factor and its interaction with the environment, including agronomic practices (Makri and Kintzios, 2008; Sgherri et al., 2010; Luz et al., 2014; Pinto et al., 2019).

The necessity to meet the growing demands of the processing industry for a clean, crunchy, uniform, tasty, and aromatic product represents a challenge for producers considering the strong effect of year-to-year variability for aromatic plants. This challenge has led the scientific community and growers to focus on alternative growing methods with controlled environmental and nutrient conditions such as hydroponics (Maggini et al., 2014; Salachas et al., 2015). These systems can guarantee higher yields, improve nutritional quality, reduce the incidence of pests and pathogens (Pardossi et al., 2006; Kiferle et al., 2013; Maboko and Du Plooy, 2013; Walters and Currey, 2015), allow the seasonal adjustment of production, and shorten production cycle (Hassanpouraghdam et al., 2010). Among hydroponic techniques, the floating raft system (FRS) is well-suited to the large-scale cultivation of relatively small medicinal and aromatic plants such as basil, due to simplicity of management and cost-effectiveness (Miceli et al., 2003; Valenzano et al., 2003; Pardossi et al., 2006; Maggini et al., 2010). Hydroponics also represents a useful method to produce leafy vegetables with a low nitrate content due to the possibility of constant monitoring of the nutrient solution (Miceli et al., 2003). The reduction of nitrates has become an important quality prerogative for the production and marketability of leafy vegetables (Orsini and De Pascale, 2007). The European Commission (EC) regulations n. 1881/2006 and 1258/2011 did not set threshold for nitrate pertaining the *Lamiaceae*. However, sweet basil can accumulate nitrate at levels higher than those permitted by the EC legislation [5,000 mg kg^–1^ of fresh weight (fw)], thus entering the hyper accumulative species (Colla et al., 2018).

In basil, different pre-harvest factors can be manipulated to improve yield and quality, leading to the conclusion that pre-harvest factors should be simultaneously analyzed to uncover their translational value and significant interactions in cultivation (Chen et al., 2004; Raimondi et al., 2006; Nicoletto et al., 2013; Corrado et al., 2020a). For instance, plant density plays a key role in shaping growth and development of different plant organs (Maboko and Du Plooy, 2013; Morano et al., 2017). Likewise, in the ordinary cultivation of basil, plants are cut more than once during the crop cycle, with harvests having a cut-specific leaf quality profile (Nicoletto et al., 2013; Corrado et al., 2020a). To the authors’ knowledge, the scientific literature has mainly focused on the manipulation of the nutrient solution to vary the qualitative and quantitative characteristics of basil in soilless systems, while evidence regarding the impact of plant density and cut, and their interaction with the genotypes, is very scarce. To fill this gap in crop science, a fully factorial experiment was conducted in hydroponics with the aim of evaluating (i) the adaptability of three Genovese basil cultivars, (ii) the impact of two densities, and (iii) the influence of two cuts on yield and quality attributes, in order to characterize and standardize production during spring season. The specific and significant morpho-physiological, phytochemicals, and aroma variations revealed the strong impact of the analyzed factors and the complexity of their interaction, whose implications are of interest also for the production of basil for the food industry.

## Materials and Methods

### Plant Material, Experimental Design, and Harvest

**FIGURE 1:**
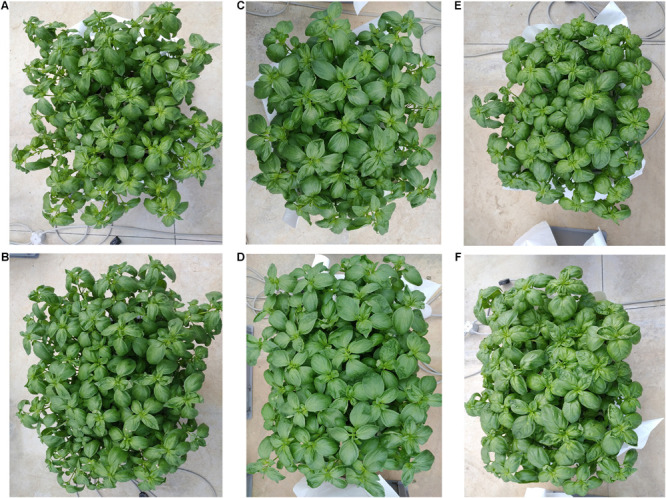
Fresh biomasses of Genovese basil plants at the end of the first harvest at different densities. **(A,B)** Illustrative pictures of Genovese basil cv. “Aroma 2” at D_*High*_ and D_*Low*_ densities. **(C,D)** Illustrative pictures of Genovese basil cv. “Eleonora” at D_*High*_ and D_*Low*_ densities. **(E,F)** Illustrative pictures of Genovese basil cv. “Italiano Classico” at D_*High*_ and D_*Low*_ densities.

The experiment was conducted at the pilot farm “Torre Lama” (Department of Agricultural Sciences, University of Naples Federico II) located in Bellizzi (SA, Italy; latitude 43°10’ N, longitude 14°58’ E, altitude 60 m a.s.l.) in a glass greenhouse with passive ventilation (10 m wide, 30 m long, 3 and 4.5 m high at the eaves and ridge, respectively) from April 11 to May 13, 2019. The mean air temperature was 25°C (min: 15°C; max: 32°C), while relative humidity was 55% during day and 79% during night. Fifteen days after sowing, seedlings of three Genovese basil (*O. basilicum* L. var. *basilicum*) cultivars “Eleonora” (Enza Zaden, Enkhuizen, Netherlands), “Aroma 2” (Fenix, Belpasso, Italy), and “Italiano Classico” (La Semiorto, Sarno, Italy) were grown in a FRS. The nutrient solution (NS) was a modified Hoagland formulation prepared with reverse osmosis water and the following nutrients: 14 mM N-NO_3_^–^, 1.75 mM S, 1.5 mM P, 3.0 mM K, 4.5 mM Ca, 1.5 mM Mg, 1.0 mM NH_4_^+^, 15 μM Fe, 9 μM Mn, 0.3 μM Cu, 1.6 μM Zn, 20 μM B, and 0.3 μM Mo. As recommended by Singh and Dunn (2016), the electrical conductivity (EC) of the NS was 2.0 ± 0.1 dS m^–1^. The pH was monitored daily and maintained at 6.0 ± 0.3 using a portable pH/EC/TDS/Temperature Meter HI991301 with HI1288 probe (Hanna instruments, Woonsocket, RI, United States). The instrument was calibrated according to the manufacturer’s recommendations with calibration solutions (two-point calibration at pH 4.01 and 7.01; EC: 1-point calibration at 12.88 dS m^–1^). The experimental design was full factorial, with three factors: cultivar (CV) with three levels (“Aroma 2,” “Eleonora,” and “Italiano Classico”), density (D) with two levels (D_*High*_ and D_*Low*_), and cut (CT) with two levels (first, CT1 and second, CT2). Each experimental unit consisted of a single plastic tank filled with 35 L of NS, containing a 54-hole polystyrene tray (52 × 32 × 6 cm; upper hole diameter: 4.5; bottom hole diameter: 3 cm; volume: 0.06 L) and an immersion pump Aquaball 60 (Eheim, Stuttgart, Germany) to maintain a constant dissolved oxygen level above the threshold limit of 6 mg L^–1^. The planting densities were 317 plant (pl) m^–2^ (54 plants/tray; D_*High*_) and 159 pl m^–2^ (27 plants/tray; D_*Low*_) (Figure 1). During the trial, basil plants were harvested twice [18 days, CT1, and 32 days after transplanting (DAT), CT2], when they reached the phenological phase of pre-flowering, leaving two internodes at CT1. Soon after CT1, the NS was replaced to guarantee the same initial mineral nutrient conditions.

### Yield, Growth, and Analysis Sampling

From each experimental unit (54 pl for D_*High*_ and 27 pl for D_*Low*_), 15 basil plants (observational unit) were sampled at each cut, separated into leaves, side branches, and stems, that were weighed and counted. Stem diameter, total fw, and leaf-to-stem ratio were recorded. A subsample of the plant was stored in paper bags and dried in a forced-air oven at 70°C until constant weight (72 h) to determine the dry weight (dw). Dry matter content was calculated as follows: dw/fw × 100. A sample of plants was collected and immediately frozen in liquid nitrogen and stored at −80°C before being freeze-dried for further qualitative analysis (i.e., phenolics and volatiles determination). For mineral determination, the dry plant material was milled and sieved with an MF10.1 Wiley laboratory mill equipped with an MF0.5 sieve (IKA, Staufen im Breisgau, Baden-Württemberg, Germany).

### CIELAB Leaf Colorimetry and Soil Plant Analysis Development (SPAD) Index

Ten colorimetric coordinates were recorded on 10 representative leaves of each experimental unit at each harvest date, using a Chroma Meter Minolta CR-300 (Minolta Co. Ltd, Osaka, Japan) calibrated with a correspondent Minolta standard. The color spaces were expressed with *L*^∗^, *a*^∗^, and *b*^∗^ values, hue angle, and chroma, as described by the International Commission of Illumination (CIE) where *L*^∗^ is degree of lightness (100) to darkness (0), *a*^∗^ is degree of greenness (−) to redness (+), and *b*^∗^ is degree of blueness (−) to yellowness (+).

Chroma and hue angle were calculated based on the following equations:

$$Chroma={\left[{\left(a*\right)}^{2}+{\left(b*\right)}^{2}\right]}^{0.5}$$

$$Hueangle=tan^{-1}\frac{b*}{a*}$$

Chroma is the “colorfulness” quantitative attribute, the degree of visual difference from neutral gray of the same lightness. A higher color intensity perceived by humans is indicated by high chroma values. The hue angle describes the qualitative color attribute in the relative amounts of redness and yellowness (i.e., the difference of certain color in reference to the gray color with the same lightness).

At 17 and 31 DAT, the SPAD index measurements as indicator of greenness were performed on 20 young fully expanded leaves of 10 representative plants per experimental unit using a portable chlorophyll meter SPAD-502 (Minolta Co. Ltd, Osaka, Japan), as described by Singh et al. (2019).

### Leaf Gas Exchange and Chlorophyll Fluorescence

At 17 and 31 DAT, between 11:00 and 13:00, gas exchange and chlorophyll fluorescence emission measurements were carried out. The measurements were performed on young fully expanded basil leaves, avoiding the central rib, using nine plants per experimental unit. The net carbon dioxide (CO_2_), assimilation rate (*A*_*CO_2*_), transpiration rate (*E*), and stomatal resistance (*r*_*s*_) were determined through a portable gas exchange analyzer (LCA 4; ADC BioScientific Ltd., Hoddesdon, United Kingdom), equipped with a broad-leaf chamber (window cuvette area of 6.25 cm^2^). The CO_2_ concentration, photosynthetically active radiation (PAR), as well as relative humidity (RH), were set to ambient values (365 ± 5 ppm, 700 ± 50 μmol photons m^–2^ s^–1^, 55 ± 5%, respectively) and the airflow rate was set to 400 ml s^–1^. The instantaneous water use efficiency (WUEi) was calculated as *A*_*CO_2*_/E.

On the same day of leaf gas exchange measurements (17 and 31 DAT), a portable fluorometer F_*v*_/F_*m*_ Meter (Opti-Sciences Inc., Hudson, United States) was used for chlorophyll fluorescence determination. Chlorophyll fluorescence was performed on the leaves of nine plants per experimental unit after their dark adaptation (for at least 10 min) by leaf clips. According to Kitajima and Butler (1975), the maximum quantum efficiency of Photosystem II (PSII) F_*v*_/F_*m*_ was calculated as (F_*m*_ - F_0_)/F_*m*_, where F_0_ and F_*m*_ were the ground fluorescence signal and the maximal fluorescence intensities in the dark-adapted state, respectively.

### Mineral Determination

The ion chromatography system ICS 3000 (Thermo Scientific Dionex, Sunnyvale, California, United States) was used to determine the cationic (K^+^, Ca^2+^, and Mg^2+^) and anionic (NO_3_^–^ and PO_4_^3–^) profile of basil, following the protocol described by Rouphael et al. (2017). For the determination of the cations, the IonPac CG12A guard column (4 × 250 mm) and the IonPac CS12A analytical column (4 × 250 mm) were used, whereas the IonPac AG11-HC guard column (4 × 50 mm) and the IonPac AS11-HC analytical column (4 × 250 mm) were used for anion determination. The ion concentrations of the tested samples were calculated based on the standard curves of cations and anions. All chemicals were purchased from Sigma-Aldrich (Milan, Italy). The detected minerals were expressed in g kg^–1^ dw, except for nitrate that was expressed in mg kg^–1^ fw by taking into consideration the dry matter percentage of each sample.

### Phenolics Determination

Phenolic extracts for high-performance liquid chromatography (HPLC) analysis were obtained following the method described by Ciriello et al. (2021), with some modifications. Briefly, 100 mg of freeze-dried basil samples was added to 2 ml of 70% aqueous methanol (*v/v*). The mixture was thoroughly mixed for 1 min (Vortex Classic stirrer; Velp Scientifica, Usmate Velate, Monza Brianza, Italy), sonicated for 20 min (Q500 ultrasonic sonicator; Qsonica, Newtown, Connecticut, United States), stirred by tilting shaker for 10 min (SSL4 see-saw rocker; Cole-Parmer, Vernon Hills, Illinois, United States), centrifuged at 6,800 rpm for 10 min (R10M, Remi Elektrotechnik Limited, Mumbai, India), and finally filtered through a 0.45-μm Teflon membrane (Phenomenex, Torrance, CA, United States). The supernatant was pipetted into a vial and analyzed by HPLC to quantify the following phenolic acids: caffeic, rosmarinic, chicoric, and ferulic acids. The chromatographic separation of phenolic acids in the extract was performed on an Agilent Technologies 1100 Series HPLC system (Palo Alto, CA, United States) equipped with a degasser (G4225A), a quaternary pump (G13111A), and a diode matrix detector (G1315B) using a 20-μl sample injection loop. A reversed-phase Kinetex C18 100-Å column (5 μm particle size, 150 × 4.6 mm; Phenomenex, Torrance, California, United States) was used. The eluents were 0.1% (*v/v*) trichloroacetic acid in water (eluent A) and acetonitrile (eluent B). The gradient schedule was 0–50% B in 50 min at a constant flow rate of 1 ml min^–1^. Identification was made by comparing the retention times with those of commercially available standards. Calibration curves were built using seven concentration levels for each standard (0.15, 0.5, 1, 10, 20, 50, and 100 mg L^–1^). The detection of each of the phenolic acids was performed at 280 nm and illustrated in Supplementary Figure 1. All HPLC grade reagents and solvents were purchased from Sigma Aldrich (Milan, Italy).

### Volatiles Determination

The extraction and quantification of volatile organic compounds (VOCs) was performed by solid-phase microextraction (SPME) and gas chromatography coupled to a mass spectrometer (GC/MS) following the protocol described by Ciriello et al. (2021). Briefly, 500 mg of fresh frozen basil was transferred into a 20-ml glass headspace vial with a Teflon septum screw cap (Supelco, Bellefonte, Pennsylvania, United States) and stirred for 10 min at 30°C (ARE magnetic stirrer; Velp Scientifica, Usmate Velate, Monza, Italy) to promote the VOCs’ migration into the headspace. A 1-cm-long and 50/30-μm-thick divinylbenzene/carboxane/polydimethylsiloxane SPME fiber (Supelco, Bellefonte, Pennsylvania, United States) was introduced into the vials for VOC adsorption. The SPME fiber was introduced into the split-splitless injector of GC 6890N coupled to MS 5973N (Agilent, Santa Clara, California, United States), where thermal desorption of the analytes was performed at 250°C for 10 min. The VOCs were separated on a 30 m × 0.250 mm capillary column coated with a 0.25-μm 5% diphenyl/95% dimethylpolysiloxane film (Supelco, Bellefonte, Pennsylvania, United States). A splitless injection was used for the samples. The temperature was maintained at 50°C for 2 min and increased from 50 to 150°C to 10°C/min and from 150 to 280°C to 15°C/min. The injection source and ion source temperatures were 250 and 230°C, respectively. Helium (99.999%) was used as the carrier gas at a 1 ml min^–1^ flow rate. The mass spectrometer was set to 70 eV. The compounds were identified using the National Institute of Standards and Technology (NIST) Atomic Spectra Database version 1.6 (U.S. Department of Commerce, Gaithersburg, Maryland, United States) and verified by retention indexes.

### Statistical Analysis

The experiment consisted of a randomized block design with three factors: Cultivar-CV, Cut-CT, and density-D. A two-way analysis of variance (ANOVA) was implemented to assess the significance of the effects and interaction between the factor pairs: CV × D, D × CT, and CV × CT. One-way ANOVA was used to compare the mean effect of CV, while CT and D were compared according to the Student’s *t*-test. Statistical significance was determined at *p* < 0.05 level using Duncan’s Multiple Range Test (DRMT) for CV × D, D × CT, and CV × CT interactions and for CV factor. All data are presented as mean ± standard error. All statistical analyses were performed using IBM SPSS 20 (Armonk, NY, United States) package for Microsoft Windows 10. Principal component analysis (PCA) was performed as described by Kassambara (2017).

## Results

### Morphological Traits and Production Response

The cultivar factor had a highly significant main effect on all the measured biometric variables, which were also strongly affected by the cut (Table 1). The lower density (D_*Low*_) resulted in a significant increase in the number of leaves, stem diameter, and number of nodes. On the other hand, the higher density (D_*High*_) led to higher fresh yield and dry biomass. The cut significantly influenced all biometric variables and, differently from the cultivar factors, there was a significant interaction effect with the density for all (but dry matter percentage) biometric variables (Table 1). For instance, a specific density × cut interaction was observed for dry biomass and leaves/stem ratio, while leaf number and fresh yield were also affected by the cultivar × density (CV × D) interaction. When the density was reduced, the leaf number increased (38.5%) while fresh yield decreased (24.1%) in all tested cultivars. Fresh yield and leaves/stem ratio were the most sensitive parameters because the three-way interaction was highly significantly. Overall, as opposed to stem diameter and leaf-to-stem ratio, the CT1 resulted in a decrease in leaf number and dry biomass for both densities. Specifically, the most significant increase in the leaf number and nodes per plant was at D_*Low*_ × CT2, which recorded the lowest stem diameter value (0.43 cm) (Table 1).

**TABLE 1:** Leaf number, stem diameter, node number, fresh yield, dry biomass, leaf-to-stem ratio, and dry matter of Genovese basil cultivars Eleonora, Aroma 2, and Italiano Classico in light of density and cut treatments.

Source of variance	Leaf number	Stem diameter	Node number	Fresh yield	Dry biomass	Leaf-to-stem ratio	Dry matter
	(no. plant^–1^)	(cm)	(no. plant^–1^)	(kg m^–2^)	(kg m^–2^)		(%)
**Cultivar (CV)**							
Eleonora	36.02 ± 3.65 b	0.44 ± 0.03 ab	3.10 ± 0.11 b	3.74 ± 0.19 c	0.43 ± 0.03 c	1.54 ± 0.03 b	11.42 ± 0.73 b
Aroma 2	43.28 ± 5.11 a	0.42 ± 0.02 b	3.43 ± 0.12 a	4.57 ± 0.35 a	0.62 ± 0.08 a	1.48 ± 0.05 c	13.13 ± 0.82 a
Italiano Classico	30.54 ± 3.40 c	0.46 ± 0.02 a	2.72 ± 0.10 c	4.40 ± 0.41 b	0.54 ± 0.06 b	1.74 ± 0.09 a	11.90 ± 0.38 b
	***	**	***	***	***	***	***
**Density (D)**							
D_*High*_	30.70 ± 2.39	0.41 ± 0.02	2.88 ± 0.09	4.81 ± 0.22	0.60 ± 0.05	1.59 ± 0.04	12.38 ± 0.58
D_*Low*_	42.52 ± 3.93	0.47 ± 0.01	3.28 ± 0.12	3.66 ± 0.26	0.46 ± 0.05	1.58 ± 0.07	11.92 ± 0.55
*t*-test	**	**	**	**	*	ns	ns
**Cut (CT)**							
CT1	25.28 ± 1.07	0.50 ± 0.01	2.92 ± 0.09	3.57 ± 0.22	0.36 ± 0.02	1.75 ± 0.05	10.14 ± 0.19
CT2	47.94 ± 2.99	0.39 ± 0.01	3.25 ± 0.12	4.91 ± 0.24	0.70 ± 0.04	1.42 ± 0.03	14.16 ± 0.37
*t*-test	***	***	*	***	***	***	***
**CV × D**							
Eleonora × D_*High*_	28.63 ± 2.20 b	0.41 ± 0.04	2.91 ± 0.14	4.15 ± 0.24 abc	0.48 ± 0.02	1.57 ± 0.05	12.03 ± 1.28
Aroma 2 × D_*High*_	37.37 ± 5.12 ab	0.39 ± 0.03	3.19 ± 0.07	5.27 ± 0.31 a	0.70 ± 0.10	1.46 ± 0.04	12.93 ± 1.20
Italiano Classico × D_*High*_	26.10 ± 3.61 b	0.42 ± 0.03	2.56 ± 0.10	5.03 ± 0.47 ab	0.62 ± 0.08	1.75 ± 0.06	12.19 ± 0.49
Eleonora × D_*Low*_	43.40 ± 5.67 ab	0.47 ± 0.04	3.30 ± 0.13	3.34 ± 0.18 c	0.37 ± 0.04	1.50 ± 0.04	10.81 ± 0.76
Aroma 2 × D_*Low*_	49.18 ± 8.64 a	0.46 ± 0.02	3.67 ± 0.20	3.88 ± 0.51 bc	0.55 ± 0.12	1.49 ± 0.10	13.34 ± 1.22
Italiano Classico × D_*Low*_	34.98 ± 5.48 ab	0.49 ± 0.01	2.88 ± 0.15	3.77 ± 0.59 bc	0.46 ± 0.09	1.74 ± 0.17	11.61 ± 0.60
	**	ns	ns	***	ns	ns	ns
**D × CT**							
D_*High*_ × CT1	22.63 ± 1.21 c	0.48 ± 0.01 b	2.83 ± 0.14 b	4.41 ± 0.12 b	0.45 ± 0.01 c	1.70 ± 0.05 a	10.20 ± 0.30
D_*Low*_ × CT1	27.92 ± 1.31 c	0.51 ± 0.01 a	3.00 ± 0.13 b	2.72 ± 0.07 c	0.27 ± 0.01 d	1.80 ± 0.08 a	10.09 ± 0.24
D_*High*_ × CT2	38.77 ± 2.54 b	0.34 ± 0.01 d	2.93 ± 0.10 b	5.21 ± 0.40 a	0.76 ± 0.06 a	1.48 ± 0.04 b	14.56 ± 0.37
D_*Low*_ × CT2	57.12 ± 3.24 a	0.43 ± 0.01 c	3.57 ± 0.15 a	4.60 ± 0.23 ab	0.64 ± 0.05 b	1.36 ± 0.02 b	13.75 ± 0.63
	***	**	**	***	*	***	ns
**CV × CT**							
Eleonora × CT1	27.35 ± 1.64 c	0.53 ± 0.01 a	3.10 ± 0.15 b	3.82 ± 0.39 b	0.35 ± 0.04 d	1.63 ± 0.03 b	9.23 ± 0.14 d
Aroma 2 × CT1	27.98 ± 0.90 c	0.47 ± 0.01 bc	3.17 ± 0.07 b	3.67 ± 0.41 b	0.38 ± 0.04 d	1.63 ± 0.04 b	10.50 ± 0.16 c
Italiano Classico × CT1	20.50 ± 1.18 c	0.49 ± 0.01 ab	2.48 ± 0.07 c	3.21 ± 0.34 b	0.35 ± 0.04 d	1.99 ± 0.06 a	10.71 ± 0.23 c
Eleonora × CT2	44.68 ± 5.10 b	0.36 ± 0.02 e	3.11 ± 0.16 b	3.67 ± 0.05 b	0.50 ± 0.02 c	1.44 ± 0.01 c	13.62 ± 0.64 b
Aroma 2 × CT2	58.57 ± 4.52 a	0.38 ± 0.02 de	3.69 ± 0.19 a	5.47 ± 0.23 a	0.86 ± 0.04 a	1.32 ± 0.02 d	15.76 ± 0.41 a
Italiano Classico × CT2	40.58 ± 3.04 b	0.42 ± 0.03 cd	2.96 ± 0.13 b	5.58 ± 0.22 a	0.73 ± 0.03 b	1.49 ± 0.06 c	13.09 ± 0.11 b
	***	***	*	***	***	***	***

ns, *, **, ***, non-significant or significant at p ≤ 0.05, 0.01, and 0.001, respectively. Different letters within each column indicate significant differences according to Duncan’s multiple-range test (p = 0.05). Density and cut factors are compared according to Student’s t-test. All data are expressed as mean ± standard error, n = 3.

### SPAD Index and Color Leaf Measurement

Significant differences were not observed among cultivars for the principal CIELAB colorimetric parameters, as opposed to SPAD index values, which were higher in “Aroma 2” and lower in “Italiano Classico” (Table 2). Both Lightness (*L*^∗^) and SPAD index showed significant variations in relation to density. D_*Low*_ density resulted in a decrease in *L*^∗^ (2.7%), in contrast to the SPAD index (+4.3%). The cut significantly influenced *b*^∗^, Chroma, and SPAD index that were reduced at CT1, in contrast to *a*^∗^ that showed an opposite trend. Significant differences were found in the interactions (CV × D, D × CT, and CV × CT) between the considered factors under investigation exclusively for SPAD index. The latter parameter increased from higher to lower density and from the first to the second cut, respectively, for CV × D and CV × CT. Specifically, the highest SPAD values were shown for D_*Low*_ × CT2 (41.96) and Aroma 2 × CT2 (43.34) (Table 2). The data indicated that the colorimetric indexes of the cultivars are fixed, as the varieties have been selected to adhere to the Genovese type standard, and little altered by the density and factors interactions.

**TABLE 2:** Soil Plant Analysis Development Index (SPAD index), CIELAB color space parameters, chroma, and hue angle of Genovese basil cultivars Eleonora, Aroma 2, and Italiano Classico in light of density and cut treatments.

Source of variance	SPAD index	L*	a*	b*	Chroma	Hue angle
**Cultivar (CV)**						
Eleonora	39.82 ± 0.53 b	41.61 ± 0.35	−7.06 ± 0.56	14.84 ± 1.22	16.44 ± 1.33	115.60 ± 0.33
Aroma 2	41.62 ± 0.60 a	41.74 ± 0.32	−7.38 ± 0.50	15.29 ± 1.19	16.98 ± 1.29	116.07 ± 0.36
Italiano Classico	38.20 ± 0.21 c	41.91 ± 0.62	−7.35 ± 0.44	16.32 ± 1.07	18.01 ± 1.14	116.99 ± 1.90
	***	ns	ns	ns	ns	ns
**Density (D)**						
D_*High*_	39.05 ± 0.40	42.32 ± 0.30	−7.32 ± 0.40	15.62 ± 0.95	17.25 ± 1.03	115.33 ± 0.27
D_*Low*_	40.71 ± 0.54	41.19 ± 0.37	−7.21 ± 0.41	15.35 ± 0.94	17.03 ± 1.02	117.11 ± 1.24
*t*-test	*	*	ns	ns	ns	ns
**Cut (CT)**						
CT1	38.88 ± 0.27	41.82 ± 0.24	−8.62 ± 0.24	18.81 ± 0.42	20.76 ± 0.43	116.31 ± 1.27
CT2	40.88 ± 0.58	41.69 ± 0.46	−5.91 ± 0.24	12.16 ± 0.56	13.53 ± 0.61	116.14 ± 0.29
*t*-test	**	ns	***	***	***	ns
**CV × D**						
Eleonora × D_*High*_	38.53 ± 0.25 b	42.02 ± 0.32	−6.74 ± 0.69	14.28 ± 1.46	15.80 ± 1.61	115.35 ± 0.51
Aroma 2 × D_*High*_	40.84 ± 0.68 a	41.84 ± 0.45	−7.35 ± 0.77	15.34 ± 1.75	17.01 ± 1.91	115.80 ± 0.32
Italiano Classico × D_*High*_	37.79 ± 0.23 b	43.09 ± 0.64	−7.87 ± 0.69	17.23 ± 1.77	18.95 ± 1.89	114.85 ± 0.55
Eleonora × D_*Low*_	41.11 ± 0.72 a	41.20 ± 0.59	−7.38 ± 0.93	15.39 ± 2.06	17.08 ± 2.26	115.86 ± 0.44
Aroma 2 × D_*Low*_	42.39 ± 0.93 a	41.64 ± 0.50	−7.41 ± 0.73	15.24 ± 1.78	16.95 ± 1.92	116.35 ± 0.65
Italiano Classico × D_*Low*_	38.61 ± 0.26 b	40.73 ± 0.86	−6.84 ± 0.54	15.42 ± 1.25	17.07 ± 1.33	119.12 ± 3.71
	**	ns	ns	ns	ns	ns
**D × CT**						
D_*High*_ × CT1	38.31 ± 0.31 b	42.21 ± 0.27	−8.80 ± 0.21	19.06 ± 0.61	21.00 ± 0.64	114.86 ± 0.30
D_*Low*_ × CT1	39.45 ± 0.36 b	41.43 ± 0.36	−8.45 ± 0.43	18.55 ± 0.59	20.51 ± 0.61	117.75 ± 2.50
D_*High*_ × CT2	39.79 ± 0.66 b	42.43 ± 0.55	−5.84 ± 0.32	12.17 ± 0.71	13.51 ± 0.77	115.80 ± 0.41
D_*Low*_ × CT2	41.96 ± 0.84 a	40.95 ± 0.67	−5.97 ± 0.38	12.15 ± 0.92	13.55 ± 0.99	116.47 ± 0.39
	*	ns	ns	ns	ns	ns
**CV × CT**						
Eleonora × CT1	38.94 ± 0.39 cd	41.51 ± 0.39	−8.77 ± 0.34	18.34 ± 0.83	20.34 ± 0.89	115.62 ± 0.24
Aroma 2 × CT1	39.90 ± 0.30 bc	41.60 ± 0.50	−8.81 ± 0.14	18.66 ± 0.47	20.64 ± 0.48	115.37 ± 0.39
Italiano Classico × CT1	37.81 ± 0.24 d	42.34 ± 0.29	−8.29 ± 0.64	19.41 ± 0.85	21.29 ± 0.88	117.93 ± 3.93
Eleonora × CT2	40.70 ± 0.89 b	41.71 ± 0.61	−5.35 ± 0.31	11.33 ± 0.95	12.54 ± 0.98	115.59 ± 0.65
Aroma 2 × CT2	43.34 ± 0.54 a	41.88 ± 0.45	−5.95 ± 0.52	11.91 ± 1.21	13.32 ± 1.32	116.78 ± 0.46
Italiano Classico × CT2	38.59 ± 0.26 cd	41.48 ± 1.25	−6.42 ± 0.32	13.24 ± 0.70	14.72 ± 0.77	116.04 ± 0.28
	***	ns	ns	ns	ns	ns

ns, *, **, ***, non-significant or significant at p ≤ 0.05, 0.01, and 0.001, respectively. Different letters within each column indicate significant differences according to Duncan’s multiple-range test (p = 0.05). Density and cut factors are compared according to Student’s t-test. All data are expressed as mean ± standard error, n = 3.

### Physiological and Biochemical Performance

The net CO_2_ assimilation rate (*A*_*CO_2*_) and the maximum quantum efficiency of open Photosystem II (F_*v*_/F_*m*_) were both affected by the cultivar (Table 3). The density choice did not affect the gas exchange parameters nor the instantaneous WUEi, but the higher density reduced F_*v*_/F_*m*_. On the other hand, the cut significantly affected all physiological measurements performed, except for WUEi. Specifically, plants harvested at CT1 showed an increase of transpiration (*E*) (17.2%) compared with CT2 and, conversely, stomatal resistance (*r*_*s*_) decreased by 24.5%. All physiological parameters were affected by the interaction between cultivar and density, revealing a robust cultivar-dependent response to the densities under investigation (Table 3). Except for F_*v*_/F_*m*_, where the lowest value was obtained at CT2 with density D_*High*_, the density × cut combination showed no difference for the physiological parameters. With respect to CV × CT, *A*_*CO_2*_ and F_*v*_/F_*m*_ showed significant differences. Particularly, “Eleonora,” and “Aroma 2” recorded the highest *A*_*CO_2*_ values at CT1, while “Eleonora” × CT2 showed the lowest F_*v*_/F_*m*_ value.

**TABLE 3:** Net photosynthesis (*A*_*CO_2_*_), stomatal resistance (*r*_*s*_), transpiration (*E*), instantaneous water use efficiency (WUEi), and chlorophyll fluorescence of Genovese basil cultivars Eleonora, Aroma 2, and Italiano Classico in light of density and cut treatments.

Source of variance	*A*_CO*_2_*_	*r*_*s*_	*E*	WUEi	Fluorescence F_*v*_/F_*m*_
	(μmol CO_2_ m^–2^ s^–1^)	(m^2^ s^–1^ mol^–1^)	(mol H_2_O m^–2^ s^–1^)	(μmol CO_2_ mol^–1^ H_2_O)	
**Cultivar (CV)**					
Eleonora	17.99 ± 0.58 b	7.44 ± 0.63	3.71 ± 0.19	4.97 ± 0.26	0.79 ± 0.01 b
Aroma 2	18.74 ± 0.45 a	6.06 ± 0.51	4.02 ± 0.21	4.79 ± 0.25	0.80 ± 0.00 a
Italiano Classico	17.60 ± 0.25 b	5.86 ± 0.86	4.15 ± 0.26	4.44 ± 0.29	0.78 ± 0.01 b
	***	ns	ns	ns	***
**Density (D)**					
D_*High*_	18.52 ± 0.35	6.13 ± 0.58	4.05 ± 0.17	4.70 ± 0.20	0.78 ± 0.01
D_*Low*_	17.70 ± 0.38	6.77 ± 0.56	3.87 ± 0.20	4.76 ± 0.25	0.80 ± 0.00
*t*-test	ns	ns	ns	ns	*
**Cut (CT)**					
CT1	19.05 ± 0.34	5.55 ± 0.25	4.28 ± 0.15	4.54 ± 0.16	0.80 ± 0.00
CT2	17.17 ± 0.25	7.35 ± 0.71	3.65 ± 0.19	4.93 ± 0.26	0.78 ± 0.01
*t*-test	***	*	**	ns	**
**CV × D**					
Eleonora × D_*High*_	19.30 ± 0.62 a	6.81 ± 0.27 ab	3.75 ± 0.08 ab	5.15 ± 0.12 ab	0.77 ± 0.01 b
Aroma 2 × D_*High*_	18.71 ± 0.63 a	4.77 ± 0.26 b	4.55 ± 0.12 a	4.11 ± 0.12 bc	0.80 ± 0.01 a
Italiano Classico × D_*High*_	17.56 ± 0.36 ab	6.80 ± 1.65 ab	3.86 ± 0.45 ab	4.83 ± 0.50 abc	0.77 ± 0.01 b
Eleonora × D_*Low*_	16.69 ± 0.64 b	8.07 ± 1.24 a	3.68 ± 0.39 ab	4.79 ± 0.52 abc	0.80 ± 0.01 a
Aroma 2 × D_*Low*_	18.76 ± 0.70 a	7.34 ± 0.64 ab	3.49 ± 0.26 b	5.47 ± 0.29 a	0.81 ± 0.00 a
Italiano Classico × D_*Low*_	17.65 ± 0.37 ab	4.91 ± 0.43 b	4.44 ± 0.26 a	4.04 ± 0.24 c	0.80 ± 0.00 a
	***	*	**	***	*
**D × CT**					
D_*High*_ × CT1	19.43 ± 0.50	5.50 ± 0.39	4.33 ± 0.23	4.58 ± 0.26	0.80 ± 0.01 a
D_*Low*_ × CT1	18.68 ± 0.47	5.60 ± 0.34	4.23 ± 0.19	4.49 ± 0.22	0.81 ± 0.00 a
D_*High*_ × CT2	17.61 ± 0.23	6.75 ± 1.08	3.77 ± 0.23	4.81 ± 0.30	0.77 ± 0.01 b
D_*Low*_ × CT2	16.73 ± 0.39	7.95 ± 0.94	3.52 ± 0.31	5.04 ± 0.44	0.80 ± 0.00 a
	ns	ns	ns	ns	*
**CV × CT**					
Eleonora × CT1	19.31 ± 0.62 a	6.03 ± 0.47	4.07 ± 0.20	4.82 ± 0.31	0.81 ± 0.00 a
Aroma 2 × CT1	20.16 ± 0.17 a	5.41 ± 0.45	4.38 ± 0.17	4.64 ± 0.19	0.81 ± 0.00 a
Italiano Classico × CT1	17.69 ± 0.39 b	5.21 ± 0.38	4.40 ± 0.37	4.15 ± 0.30	0.78 ± 0.01 b
Eleonora × CT2	16.68 ± 0.62 b	8.85 ± 0.86	3.36 ± 0.27	5.12 ± 0.44	0.76 ± 0.01 c
Aroma 2 × CT2	17.31 ± 0.21 b	6.70 ± 0.88	3.67 ± 0.34	4.94 ± 0.49	0.80 ± 0.00 ab
Italiano Classico × CT2	17.52 ± 0.34 b	6.50 ± 1.72	3.90 ± 0.38	4.73 ± 0.49	0.78 ± 0.01 b
	***	ns	ns	ns	***

ns, *, **, ***, non-significant or significant at p ≤ 0.05, 0.01, and 0.001, respectively. Different letters within each column indicate significant differences according to Duncan’s multiple-range test (p = 0.05). Density and cut factors are compared according to Student’s t-test. All data are expressed as mean ± standard error, n = 3.

### Minerals Accumulation

The effects on the mineral composition and nitrate content due to the cultivar, density, and cut are presented in Table 4. Basil cultivars affected both nitrate and assayed minerals, except for sodium. “Aroma 2” showed a lower average of nitrate (-33%) compared with the other cultivars. The lowest P and Ca content were obtained in “Eleonora” while K concentration was lower in “Italiano Classico.” Neither nitrate nor mineral composition was influenced by the density. By contrast, CT2 significantly decreased the nitrate, P, K, Ca, and Mg concentrations. Concerning the interaction between the factors under investigation, the values of nitrate and Mg were influenced by the cultivar and density. In contrast, K values were affected by the interaction between density and cut, with the lowest value obtained in D_*Low*_ × CT2 (31.13 g kg^–1^ dw). The CV × CT interaction affected Ca content, where the minimum value was obtained in “Eleonora” × CT1 (0.75 g kg^–1^ dw). However, in response to the interactions between the studied factors, P did not show substantial changes.

**TABLE 4:** Nitrate and mineral content of Genovese basil cultivars Eleonora, Aroma 2, and Italiano Classico in light of density and cut treatments.

Source of variance	Nitrate	P	K	Ca	Mg
	(mg kg^–1^ fw)	(g kg^–1^ dw)	(g kg^–1^ dw)	(g kg^–1^ dw)	(g kg^–1^ dw)
**Cultivar (CV)**					
Eleonora	3,590 ± 273 a	3.39 ± 0.37 b	41.67 ± 2.29 a	8.34 ± 0.29 b	2.51 ± 0.08 b
Aroma 2	2,332 ± 238 b	4.05 ± 0.42 a	39.31 ± 2.37 ab	9.92 ± 0.32 a	2.83 ± 0.07 a
Italiano Classico	3,418 ± 234 a	3.96 ± 0.43 a	37.28 ± 1.65 c	9.37 ± 0.44 a	2.45 ± 0.13 b
	***	***	**	***	***
**Density (D)**					
D_*High*_	2,872 ± 189	3.73 ± 0.35	39.84 ± 1.20	9.00 ± 0.34	2.66 ± 0.07
D_*Low*_	3,354 ± 272	3.87 ± 0.32	39.00 ± 2.19	9.42 ± 0.30	2.54 ± 0.10
*t*-test	ns	ns	ns	ns	ns
**Cut (CT)**					
CT 1	3,785 ± 174	5.12 ± 0.11	45.30 ± 1.05	10.08 ± 0.29	2.78 ± 0.07
CT 2	2,442 ± 182	2.48 ± 0.09	33.54 ± 1.04	8.34 ± 0.21	2.41 ± 0.08
*t*-test	***	***	***	***	***
**CV × D**					
Eleonora × D_*High*_	3,156 ± 410 ab	3.31 ± 0.57	40.13 ± 2.69	7.76 ± 0.27	2.40 ± 0.06 bc
Aroma 2 × D_*High*_	2,339 ± 100 b	4.08 ± 0.62	40.65 ± 1.26	9.98 ± 0.57	2.93 ± 0.09 a
Italiano Classico × D_*High*_	3,122 ± 317 ab	3.79 ± 0.68	38.73 ± 2.31	9.25 ± 0.54	2.64 ± 0.12 abc
Eleonora × D_*Low*_	4,025 ± 289 a	3.47 ± 0.52	43.20 ± 3.87	8.91 ± 0.40	2.63 ± 0.13 abc
Aroma 2 × D_*Low*_	2,324 ± 489 b	4.02 ± 0.62	37.97 ± 4.74	9.85 ± 0.36	2.72 ± 0.08 ab
Italiano Classico × D_*Low*_	3,714 ± 323 a	4.13 ± 0.60	35.84 ± 2.42	9.48 ± 0.74	2.27 ± 0.21 c
	**	ns	ns	ns	**
**D × CT**					
D_*High*_ × CT1	3,445 ± 240	5.10 ± 0.16	43.73 ± 0.84 a	9.88 ± 0.49	2.78 ± 0.11
D_*Low*_ × CT1	4,125 ± 207	5.13 ± 0.16	46.87 ± 1.84 a	10.27 ± 0.32	2.78 ± 0.09
D_*High*_ × CT2	2,300 ± 110	2.35 ± 0.11	35.94 ± 1.26 b	8.11 ± 0.26	2.53 ± 0.08
D_*Low*_ × CT2	2,584 ± 352	2.61 ± 0.13	31.13 ± 1.24 c	8.56 ± 0.32	2.30 ± 0.13
	ns	ns	***	ns	ns
**CV × CT**					
Eleonora × CT1	4,279 ± 174	4.58 ± 0.10	48.31 ± 1.95	8.83 ± 0.43 bc	2.65 ± 0.12
Aroma 2 × CT1	2,953 ± 224	5.42 ± 0.13	45.81 ± 1.36	10.65 ± 0.39 a	2.94 ± 0.11
Italiano Classico × CT1	4,123 ± 142	5.35 ± 0.13	41.79 ± 1.16	10.74 ± 0.23 a	2.74 ± 0.11
Eleonora × CT2	2,902 ± 328	2.20 ± 0.12	35.02 ± 1.28	7.84 ± 0.30 d	2.37 ± 0.05
Aroma 2 × CT2	1,710 ± 211	2.68 ± 0.06	32.82 ± 2.47	9.18 ± 0.30 b	2.71 ± 0.05
Italiano Classico × CT2	2,714 ± 146	2.57 ± 0.18	32.77 ± 1.60	8.00 ± 0.20 cd	2.16 ± 0.16
	ns	ns	ns	*	ns

ns, *, **, ***, non-significant or significant at p ≤ 0.05, 0.01, and 0.001, respectively. Different letters within each column indicate significant differences according to Duncan’s multiple-range test (p = 0.05). Density and cut factors are compared according to Student’s t-test. All data are expressed as mean ± standard error, n = 3.

### Quantification of Phenolic Acids

Total phenolic acids were affected by the factors under investigation and their interactions (Table 5). Rosmarinic acid was the most prevalent compound, followed by chicoric, caffeic, and ferulic acids. “Italiano Classico” showed the highest content of rosmarinic (144.0 μg g^–1^ dw) and chicoric acids (74.49 μg g^–1^ dw) with an overall higher accumulation of 44.2% (on average) in total phenolic acids, compared to the other two cultivars. The density influenced the content of the most abundant phenolic acids (rosmarinic and chicoric acids), as well as the total phenolic acids content. Except for rosmarinic acid, the cut impacted all the phenolic profile. In addition, the interaction between cultivar and density affected rosmarinic, chicoric, and caffeic acids including total phenolic acids. Moreover, for all cultivars, D_*Low*_ density led to an increase of rosmarinic, chicoric, and total phenolic acids by 58.3, 84.2, and 55.2%, respectively. In addition, the concentration of all phenolic acids and their sum (total phenolic acids) was affected by the density × cut interaction with D_*Low*_ × CT2 combination resulting in their highest accumulation. Lastly, the phenolic profile was strongly affected by CV × CT, increasing from the first to the second cut for all the studied cultivars.

**TABLE 5:** Phenolic acids and total polyphenols of Genovese basil cultivars Eleonora, Aroma 2, and Italiano Classico in light of density and cut treatments.

Source of variance	Caffeic acid	Chicoric acid	Rosmarinic acid	Ferulic acid	Total phenolic acids
	(μg g^–1^ dw)	(μg g^–1^ dw)	(μg g^–1^ dw)	(μg g^–1^ dw)	(μg g^–1^ dw)
**Cultivar (CV)**					
Eleonora	40.94 ± 3.55 b	56.59 ± 8.21 c	46.75 ± 3.49 c	4.63 ± 0.72 a	145.5 ± 11.9 c
Aroma 2	55.69 ± 8.77 a	67.35 ± 12.0 b	111.9 ± 16.6 b	4.88 ± 0.64 a	237.9 ± 35.5 b
Italiano Classico	55.51 ± 1.70 a	74.49 ± 19.5 a	144.0 ± 12.6 a	3.24 ± 0.38 b	276.4 ± 32.3 a
	***	***	***	***	***
**Density (D)**					
D_*High*_	46.71 ± 3.21	46.55 ± 6.09	78.15 ± 9.62	3.83 ± 0.44	172.4 ± 12.8
D_*Low*_	54.72 ± 5.77	85.74 ± 13.4	123.7 ± 15.3	4.53 ± 0.52	267.5 ± 31.3
*t*-test	ns	*	*	ns	**
**Cut (CT)**					
CT1	39.76 ± 2.77	35.28 ± 2.45	85.10 ± 10.4	2.67 ± 0.13	159.8 ± 12.9
CT2	61.66 ± 4.87	97.01 ± 12.0	116.7 ± 15.7	5.50 ± 0.44	279.8 ± 28.7
*t*-test	***	***	ns	***	***
**CV × D**					
Eleonora × D_*High*_	48.68 ± 5.16 bc	47.27 ± 2.21 b	38.89 ± 4.09 b	3.91 ± 0.40	135.3 ± 10.7 c
Aroma 2 × D_*High*_	36.21 ± 6.20 c	59.49 ± 17.3 b	72.24 ± 5.70 b	5.09 ± 0.80	173.0 ± 29.8 c
Italiano Classico × D_*High*_	55.23 ± 1.56 b	32.91 ± 1.91 b	123.3 ± 13.1 a	2.54 ± 0.13	212.1 ± 10.7 bc
Eleonora × D_*Low*_	33.21 ± 2.20 c	65.92 ± 16.0 ab	54.61 ± 3.47 b	4.98 ± 1.06	158.7 ± 21.7 c
Aroma 2 × D_*Low*_	75.17 ± 12.2 a	75.22 ± 17.8 ab	151.6 ± 23.5 a	4.67 ± 1.06	306.7 ± 53.5 ab
Italiano Classico × D_*Low*_	55.78 ± 3.19 b	116.1 ± 31.4 a	164.7 ± 19.0 a	3.95 ± 0.65	340.5 ± 53.5 a
	***	***	***	ns	***
**D × CT**					
D_*High*_ × CT1	37.46 ± 4.40 c	33.17 ± 4.24 c	79.64 ± 18.2 b	2.91 ± 0.25 c	152.2 ± 20.8 b
D_*Low*_ × CT1	42.07 ± 3.43 bc	37.40 ± 2.51 bc	90.56 ± 11.2 b	2.51 ± 0.11 c	171.3 ± 16.6 b
D_*High*_ × CT2	55.95 ± 1.71 ab	59.94 ± 9.73 b	76.67 ± 7.87 b	4.44 ± 0.64 b	195.8 ± 12.0 b
D_*Low*_ × CT2	67.37 ± 9.47 a	134.1 ± 13.3 a	156.8 ± 24.3 a	6.55 ± 0.36 a	363.8 ± 40.3 a
	**	***	***	***	***
**CV × CT**					
Eleonora × CT1	33.05 ± 2.53 c	39.56 ± 4.34 b	39.87 ± 4.89 b	2.69 ± 0.21 b	113.8 ± 4.46 c
Aroma 2 × CT1	35.28 ± 5.74 c	28.44 ± 3.49 b	79.83 ± 8.88 b	2.88 ± 0.26 b	144.1 ± 18.5 bc
Italiano Classico × CT1	50.96 ± 0.70 bc	37.85 ± 3.96 b	135.6 ± 9.12 a	2.46 ± 0.14 b	225.4 ± 7.82 b
Eleonora × CT2	48.84 ± 4.90 bc	73.63 ± 12.7 ab	53.63 ± 3.27 b	5.59 ± 0.81 a	180.2 ± 11.2 bc
Aroma 2 × CT2	76.10 ± 11.8 a	106.3 ± 4.59 a	144.0 ± 26.9 a	6.88 ± 0.35 a	331.8 ± 43.1 a
Italiano Classico × CT2	60.05 ± 1.98 ab	111.1 ± 33.5 a	152.5 ± 24.3 a	4.03 ± 0.61 b	327.7 ± 59.0 a
	***	***	***	***	***

ns, *, **, ***, non-significant or significant at p ≤ 0.05, 0.01, and 0.001, respectively. Different letters within each column indicate significant differences according to Duncan’s multiple-range test (p = 0.05). Density and cut factors are compared according to Student’s t-test. All data are expressed as mean ± standard error, n = 3.

### Volatile Profile Estimation

The percentages of the major volatile compounds are shown in Table 6. Linalool was the most prevalent compound, followed by eucalyptol, eugenol, α-bergamotene, 1-octen-3-ol, and β-cis-ocimene. Except for eucalyptol, all volatile compounds detected were affected significantly by the cultivar. “Eleonora” recorded the highest concentration of 1-octen-3-ol and α-bergamotene but the lowest linalool concentration; instead, “Italiano Classico” showed the lowest β-cis-ocimene value while “Aroma 2” showed the lowest eugenol percentage. The density only influenced the β-cis-ocimene content, with the highest value recorded in D_*Low*_. Conversely, all volatile compounds, except β-cis-ocimene, were affected by the cut. In contrast to linalool, eugenol, and α-bergamotene, the highest percentage values of eucalyptol and 1-octen-3-ol were obtained at the second cut. 1-octen-3-ol, β-cis-ocimene, and linalool buildup were influenced exclusively by the interaction between cultivar and density, with the latter exhibiting the lowest value in “Eleonora” × D_*Low*_ (36.1%). The interaction between the density and cut showed significant variations for eucalyptol, linalool, and α-bergamotene. Specifically, eucalyptol content was higher in D_*High*_ × CT2 (31.1%). Interaction between cultivar and cut resulted in differences exclusively for eucalyptol and α-bergamotene content, with the latter showing the maximum value in “Eleonora” × CT1.

**TABLE 6:** Most abundant volatile compounds of Genovese basil cultivars Eleonora, Aroma 2, and Italiano Classico in light of density and cut treatments.

Source of variance	1-Octen-3-ol	Eucalyptol	β -cis-Ocimene	Linalool	Eugenol	α -Bergamotene
	(%)	(%)	(%)	(%)	(%)	(%)
**Cultivar (CV)**						
Eleonora	4.03 ± 0.05 a	25.72 ± 1.45	3.09 ± 0.15 a	38.49 ± 1.01 b	4.59 ± 0.22 a	5.17 ± 0.56 a
Aroma 2	2.86 ± 0.11 c	25.71 ± 1.83	2.97 ± 0.30 a	44.56 ± 0.87 a	3.92 ± 0.25 b	3.13 ± 0.35 b
Italiano Classico	3.30 ± 0.13 b	25.58 ± 0.63	2.36 ± 0.29 b	44.84 ± 0.94 a	4.51 ± 0.29 a	2.97 ± 0.16 b
	***	ns	***	***	**	***
**Density (D)**						
D_*High*_	3.47 ± 0.13	26.90 ± 1.31	2.19 ± 0.17	43.32 ± 0.74	4.17 ± 0.23	3.51 ± 0.47
D_*Low*_	3.32 ± 0.15	24.44 ± 0.80	3.42 ± 0.15	41.94 ± 1.24	4.51 ± 0.20	4.01 ± 0.29
*t*-test	ns	ns	***	ns	ns	ns
**Cut (CT)**						
CT1	3.17 ± 0.15	22.73 ± 0.75	2.84 ± 0.17	44.14 ± 1.03	5.03 ± 0.13	4.55 ± 0.43
CT2	3.62 ± 0.11	28.61 ± 0.97	2.77 ± 0.26	41.13 ± 0.91	3.65 ± 0.14	2.97 ± 0.25
*t*-test	*	***	ns	*	***	**
**CV × D**						
Eleonora × D_*High*_	3.99 ± 0.09 a	26.40 ± 2.92	2.81 ± 0.15 bc	40.88 ± 1.15 b	4.55 ± 0.34	5.22 ± 1.06
Aroma 2 × D_*High*_	2.91 ± 0.17 c	27.67 ± 2.84	2.19 ± 0.34 cd	45.98 ± 1.10 a	3.57 ± 0.35	2.66 ± 0.47
Italiano Classico × D_*High*_	3.51 ± 0.13 b	26.63 ± 0.81	1.57 ± 0.10 d	43.11 ± 0.70 ab	4.40 ± 0.41	2.63 ± 0.18
Eleonora × D_*Low*_	4.07 ± 0.05 a	25.05 ± 0.73	3.37 ± 0.21 ab	36.11 ± 0.94 c	4.63 ± 0.31	5.11 ± 0.51
Aroma 2 × D_*Low*_	2.81 ± 0.16 c	23.75 ± 2.26	3.75 ± 0.21 a	43.14 ± 1.15 ab	4.27 ± 0.32	3.60 ± 0.47
Italiano Classico × D_*Low*_	3.09 ± 0.20 c	24.52 ± 0.81	3.14 ± 0.33 ab	46.58 ± 1.48 a	4.62 ± 0.44	3.31 ± 0.18
	*	ns	***	***	ns	ns
**D × CT**						
D_*High*_ × CT1	3.28 ± 0.19	22.70 ± 1.09 c	2.46 ± 0.19 b	44.59 ± 1.06	4.88 ± 0.18	4.63 ± 0.76 a
D_*Low*_ × CT1	3.05 ± 0.24	22.76 ± 1.10 c	3.22 ± 0.23 a	43.69 ± 1.83	5.18 ± 0.20	4.47 ± 0.45 a
D_*High*_ × CT2	3.66 ± 0.16	31.10 ± 1.30 a	1.92 ± 0.26 b	42.06 ± 0.92	3.46 ± 0.24	2.39 ± 0.24 b
D_*Low*_ × CT2	3.59 ± 0.16	26.11 ± 0.88 b	3.62 ± 0.18 a	40.20 ± 1.58	3.83 ± 0.14	3.55 ± 0.34 ab
	ns	***	***	ns	ns	**
**CV × CT**						
Eleonora × CT1	3.92 ± 0.06	21.92 ± 0.89 cd	3.25 ± 0.26	40.17 ± 1.34	5.20 ± 0.07	6.67 ± 0.49 a
Aroma 2 × CT1	2.52 ± 0.04	20.17 ± 0.82 d	3.17 ± 0.18	46.23 ± 1.29	4.63 ± 0.17	4.05 ± 0.36 b
Italiano Classico × CT1	3.06 ± 0.17	26.11 ± 0.77 b	2.10 ± 0.20	46.01 ± 1.62	5.27 ± 0.32	2.92 ± 0.13 bc
Eleonora × CT2	4.14 ± 0.06	29.52 ± 1.64 a	2.93 ± 0.13	36.82 ± 1.26	3.98 ± 0.24	3.67 ± 0.50 b
Aroma 2 × CT2	3.20 ± 0.09	31.25 ± 1.33 a	2.77 ± 0.60	42.90 ± 0.76	3.21 ± 0.21	2.21 ± 0.24 c
Italiano Classico × CT2	3.53 ± 0.15	25.04 ± 1.03 bc	2.61 ± 0.55	43.67 ± 0.84	3.75 ± 0.19	3.02 ± 0.31 bc
	ns	***	ns	ns	ns	***

ns, *, **, ***, non-significant or significant at p ≤ 0.05, 0.01, and 0.001, respectively. Different letters within each column indicate significant differences according to Duncan’s multiple-range test (p = 0.05). Density and cut factors are compared according to Student’s t-test. All data are expressed as mean ± standard error, n = 3.

### Principal Component Analysis

**FIGURE 2:**
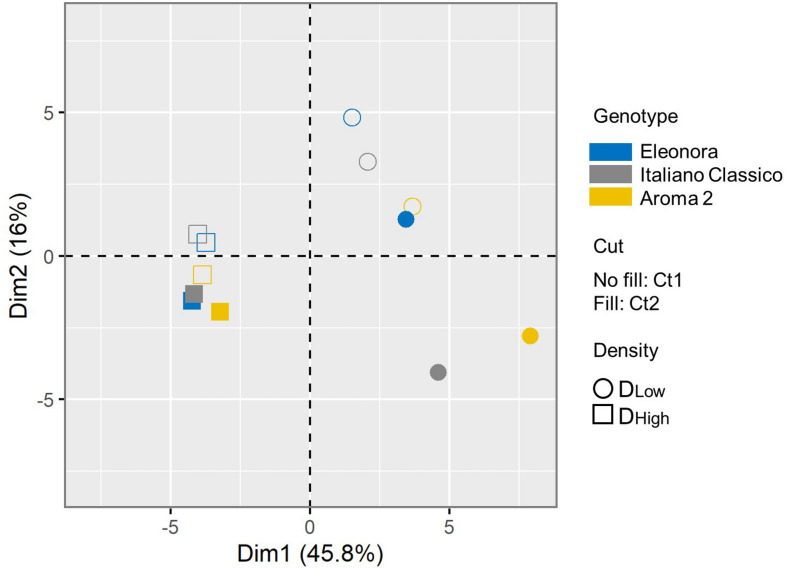
Principal component analysis (PCA) of the basil response. Symbol shape indicates the growing density (D_*Low*_: circle; D_*High*_: square). Each condition is colored according to the variety (“Eleonora”: blue; “Italiano Classico”: gray; “Aroma 2”: gold). For each cut, the plot displays the symbol empty (CT1) or filled (CT2). The color and symbol legends are reported on the right side.

A PCA was conducted for all the agronomical and physicochemical composition parameters assessed in this study, which were shaped by the investigated factors and their significant interactions. The first two components accounted for 61.8% of the total variance (Supplementary Figure 2). The two-dimensional component plot uncovered an internal structure of the data consistent with the experimental factors (Figure 2). Samples were separated coherently along the PC1 based on the density, with all D_*Low*_ samples (respectively, D_*High*_) in the positive (resp, negative) PC1 plot area. Considering the prominent contribution of the first component (45.8% of total variance), the density factor associated with the largest linearly projected variance in the measured basil traits. Moreover, samples were much more distributed at the lower planting density, indicating that the total common variance of the basil traits is restrained when plants grow tighter. Considering the cut, there was good separation along the PC2 for nearly all samples. The clustering of the samples according to the cultivar indicated that the genotype-dependent effect on the measured traits does not vary strongly depending on the conditions, and it is inferior to the other pre-harvest factors, as the three varieties consistently clustered according to the level of the other two factors (cut and density). It should be added that PCA orthogonally transforms data, and the grouping of the cultivars may also be interpreted considering a possible non-linear genotypic-dependent response to the cut and density of the different varieties. Overall, the multivariate analysis indicated that most of the variance can be explained considering the two growing conditions, and that, at higher density, the variability of the measured traits due to the genotype and cut factors is less extensive.

## Discussion

The FRS is a valuable tool to deseasonalize, anticipate, and improve basil plants’ productivity, useful also to understand plant response to the combined action of different pre-harvest factors on various classes of basil traits. The biometric parameters were the most affected, followed by polyphenols, considering the relative presence of three-way interactions. From an applied perspective, it is noteworthy that the fresh biomass per area was affected by each factor and all their interactions. Among the yield components, the number of leaves was the highly sensitive parameter to the various factors and interaction. Also, total polyphenols were highly affected by all the factors and this is reasonable considering their inducible accumulation and, as indicated by our data, that distinct major polyphenols of sweet basil vary differently according to the pre-harvest factors.

Our data showed an improved production performance of the tested cultivars, both in fresh yield and in advance production, achieving yields about twofold higher than those obtained by Nicoletto et al. (2013) in the open field. Regardless of plant density and cuts, “Aroma 2” exhibited a better adaptability to the FRS, ensuring higher fresh yield and dry biomass per square meter, which can be ascribed to a better photosynthetic performance and a higher number of leaves and nodes per plant. On the contrary, a recent comparative study illustrated for the same cultivars grown in the autumn–winter season a diametrically opposite production response, indicating a high impact of the environmental factors (Ciriello et al., 2020).

Apart from plant material, both the cut and density affected yield and yield-related parameters. Similar to Zheljazkov et al. (2008) and Puccinelli et al. (2021), a linear increase in fresh yield, dry biomass, number of leaves, and nodes per plant were marked from the first to the second cut. As suggested by Zheljazkov et al. (2008), the increase in production could be due to a well-formed root system at the second cut that facilitates a faster regrowth of the epigeal part. Moreover, the suppression of apical dominance would have stimulated lateral buds’ emission, which led to an increase in the number of nodes and leaves per plant, and consequently to a decrease in the leaf-to-stem ratio (Tekalign and Hammes, 2005). Other studies on herbaceous crop suggested that the cut may increase cytokinin concentration, hence stimulating cell division and regulating the leaf primordia emission (Le Bris, 2017; Skalák et al., 2019).

Prior to the second harvest, gas exchange measurements showed a decrease of plants’ main physiological parameters, such as transpiration rate, net CO_2_ fixation, and increased stomatal resistance compared to CT1. These results could be attributed to the combined effects of cut and tissues lignification of plant nearing the end of their life cycle. Scientific evidence demonstrated a direct relationship between the end of life cycle and the reduction in the photosynthetic activity, attributed to a degradation of RuBisCO activity and the alteration of redox processes involving the electron transport chain (Krieger-Liszkay et al., 2019). The minimal reduction in net CO_2_ assimilation rate, transpiration, and F_*v*_/F_*m*_ ratio would confirm the onset of leaf senescence processes in the plants at the second cut. The observed phenomenon was also confirmed by the increase in dry matter, due to the progressive lignification of plant tissues (Corrado et al., 2020b). Noteworthy, for the industrial processing of pesto, the dry matter content is a crucial technological parameter. An excessive fibrousness would extend the processing duration, thus causing oxidation with a decrease in the quality of the final product (pesto blackening) (Nicoletto et al., 2013). Another crucial industrial requirement is basil leaves’ color, which drives consumer choice (León et al., 2006). Colorimetric parameters were not affected by genotype, like the results obtained in a recent open field trial wherein the same cultivars were compared for production and quality (Ciriello et al., 2021). However, the cut resulted in a reduction in perceived color intensity (Chroma), attributable to both *a*^∗^ and *b*^∗^ variations, probably due to the lower nitrate content in basil leaves (Fallovo et al., 2009).

On the other hand, density choice did not affect food processing key parameters such as dry matter and leaf-to-stem ratio, in contrast to the observations of Miceli et al. (2003), which reported an increase in dry matter with density growth. This result can be attributed to the different plant material and the different densities that were almost double (226 and 593 plants m^–2^) compared to those tested (159 and 317 plants m^–2^) in the current study. However, the double density (D_*High*_) in our experiment led to an increased fresh yield and dry shoot biomass for all assayed cultivars, as supported by the results reported in the reviewed literature (Miceli et al., 2003; Maboko and Du Plooy, 2013; Mahlangu et al., 2020). Nonetheless, the increased fresh yield and dry biomass at the higher density is due to the higher number of plants per unit area (Maboko and Du Plooy, 2013), as highlighted by the lower number of leaves and nodes per plant. It should be added that in hydroponics, neighboring plants little compete for below−ground resources (water and nutrients). The reduction in the number of nodes is probably caused by the lower light capture of the canopy because the resources competition increases with the distance decrease (Postma et al., 2020). An interesting study by Ballaré and Pierik (2017) revealed that plants grown at high densities, due to a reduced ratio between red and far-red light (R:FR) in the canopy, reduce the diameter of the stem, corroborating our findings.

Our results showed a significant cultivar-dependent response for mineral accumulation, in agreement with the findings of Licina et al. (2014), who compared the mineral composition of different basil genotypes. The positive lower nitrate accumulation recorded in “Aroma 2” emphasizes the genotype’s key role in accumulating this potentially risky dietary compound for human health (Colla et al., 2018). This may be connected to a different expression of genes involved in nitrate transport, as shown in lettuce (Razgallah et al., 2017) and/or a higher nitrate reductase activity (Luo et al., 2006). Magnesium is a central cation of the chlorophyll molecule and involved in RuBisCO activation, promoting CO_2_ assimilation (Karthika et al., 2018). The higher magnesium content in “Aroma 2” is reflected in the higher SPAD and net CO_2_ assimilation values, which resulted in higher fresh yield. In contrast to the density effect, successive cuts resulted in a decrease in all analyzed minerals. However, the overall mineral profile reduction was associated with a significant increase in dry matter (about twice as much) from the first to the second cut. This would explain the decrease in minerals as an effect of dilution and not directly attributable to cut-induced distress (Jarrell and Beverly, 1981).

Besides synthesizing primary compounds for growth and development, plants produce a wide range of specialized metabolites, such as phenolics, which act as passive defense barriers (Trivellini et al., 2016). Their biosynthesis is strongly affected by genotype and environmental stressors (Rouphael et al., 2012). As outlined in our investigation, phenolic acids were strongly influenced by genotype. “Aroma 2” and “Italiano Classico” phenolic profiles had a higher concentration of rosmarinic acid (a compound found to be the more predominant in basil), in contrast to “Eleonora” that accumulated more chicoric acid. A recent study performed in an FRS provided comparable results, highlighting a significant cultivar-dependent response to chicoric and rosmarinic acid accumulation using the same cultivars of Genovese basil (Ciriello et al., 2020). Rosmarinic acid accumulation was higher than the one obtained by Sgherri et al. (2010) in a soilless experiment, but well below the values of Javanmardi et al. (2002) in the open field. These discrepancies can be ascribed to the different growing conditions, extraction and determination methods, and various plant material adopted by each author (Filip, 2017). A study carried out by Kwee and Niemeyer (2011) revealed in the spice basil (*O. basilicum* × *O. americanum*) a lower content of chicoric acid compared to our findings. In contrast, Thai basil (*O. basilicum* var. *thyrsiflorum*) had a higher chicoric acid content, underlining the impact of genotype on biosynthesis and accumulation of phenolic acids. Concerning the total phenolic acid content, this study showed values about fourfold lower than those obtained by the same cultivars in an open field experiment (Ciriello et al., 2021). The higher values obtained in the open field may be imputable to pedoclimatic conditions, less favorable than those in the soilless system, leading to an oxidative stress that fostered phenolic acids accumulation as a defense mechanism (Sgherri et al., 2010). Furthermore, continuous exposure of field-grown plants to UV radiation can prompt higher phenylalanine ammonia-lyase (PAL) activity resulting in increased phenolic acid accumulation (Neocleous and Ntatsi, 2018; Loconsole and Santamaria, 2021). Additionally, specialized metabolite biosynthesis is also influenced by perceived solar radiation, varying with seasonality and planting density. Therefore, the rise of total phenolic acids with the lowest density (D_*Low*_) could be due to a lower shading of the plants. Apart from having a positive effect on primary metabolism, light is a critical parameter for producing carbon compounds in plants such as phenolic acids (Kumar et al., 2013). Similarly, the accumulation of phenolic acids is stimulated by stress factors that cause the evolution of “reactive oxygen species (ROS)” in plant tissues (Naikoo et al., 2019). Like other biotic and abiotic stresses, the cut led to a linear increase in the total phenolic acid content in sweet basil, as confirmed by Nicoletto et al. (2013) and Ciriello et al. (2021). The increase in total phenolic acids in response to cut suggests that this agronomic practice might promote PAL activity; in addition, better production performance at the second harvest might have led to an increased allocation of photosynthates to the shikimic acid pathway (Shaw et al., 1998; Crozier et al., 2007).

Basil is also endowed with aromatic molecules belonging to different chemical groups (i.e., monoterpenes, sesquiterpenes, and phenylpropanoids), whose composition confers the characteristic aroma and taste of the plant (Salvadeo et al., 2007). The tested cultivars showed either the absence of undesirable aromatic compounds (e.g., estragole, thymol, and carvacrol) or a predominance (more than 60%) of oxygenated monoterpenes such as linalool and eucalyptol, typical volatiles of Genovese cultivars used for pesto sauce production (Salvadeo et al., 2007). Variations in volatiles composition among cultivars were attributable to the different percentage content of minor aromatic compounds, mainly related to different genotypes’ intrinsic characteristics (Ibrahim et al., 2006). The higher concentration of 1-octen-3-ol and α-bergamotene in “Eleonora” and the lower of β-cis-Ocimene in “Italiano Classico” are traits fixed by the genotype. Recent experiments carried out under different conditions and growth systems with the same cultivars showed an increased accumulation of the abovementioned minor compounds, which contribute to enrich and diversify the aromatic bouquet of basil (Ciriello et al., 2020, 2021). In dill (*Anethum graveolens* L.) plants grown in the open field, the employment of high densities resulted in significantly increased amounts of major aroma compounds due to the root competition for water and nutrients (El-Zaeddi et al., 2017). However, in our experiment, independently from the cultivar, the density choice did not induce significant variations in eucalyptol and linalool values. On the other hand, the aroma profile of basil changed in response to successive cuts. In agreement with Ciriello et al. (2021), the cut significantly impacted the expression of the major volatiles (eucalyptol and linalool), thus confirming the strict link between the volatiles’ biosynthesis and stressors. However, concerning the results of several open field trials, the second cut reduced the linalool content (Zheljazkov et al., 2008; Tsasi et al., 2017; Ciriello et al., 2021). This difference could be attributed either to using different growing systems (open field vs. FRS) or the different climatic conditions that characterized the experiments (Luz et al., 2014). In contrast to linalool content, eucalyptol increased significantly at the second cut; probably, the cut induced a better expression of the enzyme 1,8-cineole synthase, which converts geranyl pyrophosphate (GPP) to eucalyptol, at the expense of the enzyme linalool synthase (LIS), which catalyzes the GPP-Linalool reaction (Chang et al., 2007). Apart from the factors under investigation, the cut caused a decrease in eugenol as observed in an open field study on basil (Tsasi et al., 2017). Similarly, research on sorrel (*Rumex acetosa* L.) showed a significant reduction of sesquiterpenes concentration, evidenced by the reduced α-bergamotene at the second cut (Ceccanti et al., 2020).

## Conclusion

The increased demand of the food industry for fresh basil with standardized technological and aromatic attributes has fostered the diffusion of hydroponics. Among the tested cultivars, “Aroma 2” ensured the best production performance, the lowest nitrate content, and the highest dry matter percentage. The latter, as well as the aromatic profile, were not affected by the density, whereas the yield was increased with the highest density. Successive cuts, ordinarily performed for basil production, also increased the yield per area and favored the accumulation of phenolic acids (+75.1%), without modifying linalool content, though triggering eucalyptol (+25.9%) and 1-octen-3-ol (+15.1%) accumulation. Our work provides useful information on the productive and qualitative response of the main basil cultivars used for the food industry. The observed wide-ranging responsiveness also suggests that an assessment under different climatic conditions (e.g., autumn cycle) will be a useful complement to manage the year-round production of Genovese leaves for the food industry. Finally, future research may also explore the here described impact of the cut on the phenolic acids’ accumulation as a possible fortification means to extend the pesto sauce shelf life, reducing the need of added antioxidants and thermal processing.

## Data Availability Statement

The raw data supporting the conclusions of this article will be made available by the authors, without undue reservation.

## Author Contributions

YR: conceptualization and project administration. MC and LF: methodology, validation, formal analysis, investigation, and writing—original draft preparation. AP: software. YR and SDP: resources. MC, LF, and AP: data curation. MC, LF, CE-N, GC, and YR: writing—review and editing. GC and YR: visualization. GC, SDP, and YR: supervision. SDP: funding acquisition. All authors contributed to the article and approved the submitted version.

## Conflict of Interest

The authors declare that the research was conducted in the absence of any commercial or financial relationships that could be construed as a potential conflict of interest.

## Supplementary Material

Supplementary Figure 1 Chromatograms of phenolic acids in Genovese basil extract by HPLC at density D2 with separation of caffeic acid (1), ferulic acid (2), chicoric acid (3), and rosmarinic acid (4). **(A,B)** Aroma 2 at first and second cut. **(C,D)** Eleonora at first and second cut. **(E,F)** Italiano Classico at first and second cut.

Supplementary Figure 2 Scree plot of the eigenvalues of the principal components.
