# Supplementary figures and images for: Morpho-Physiological Responses and Secondary Metabolites Modulation by Preharvest Factors of Three Hydroponically Grown Genovese Basil Cultivars
Source: Front Plant Sci. 2021 Apr 26;12:671026. doi: 10.3389/fpls.2021.671026 (PMC8107287; doi:10.3389/fpls.2021.671026)

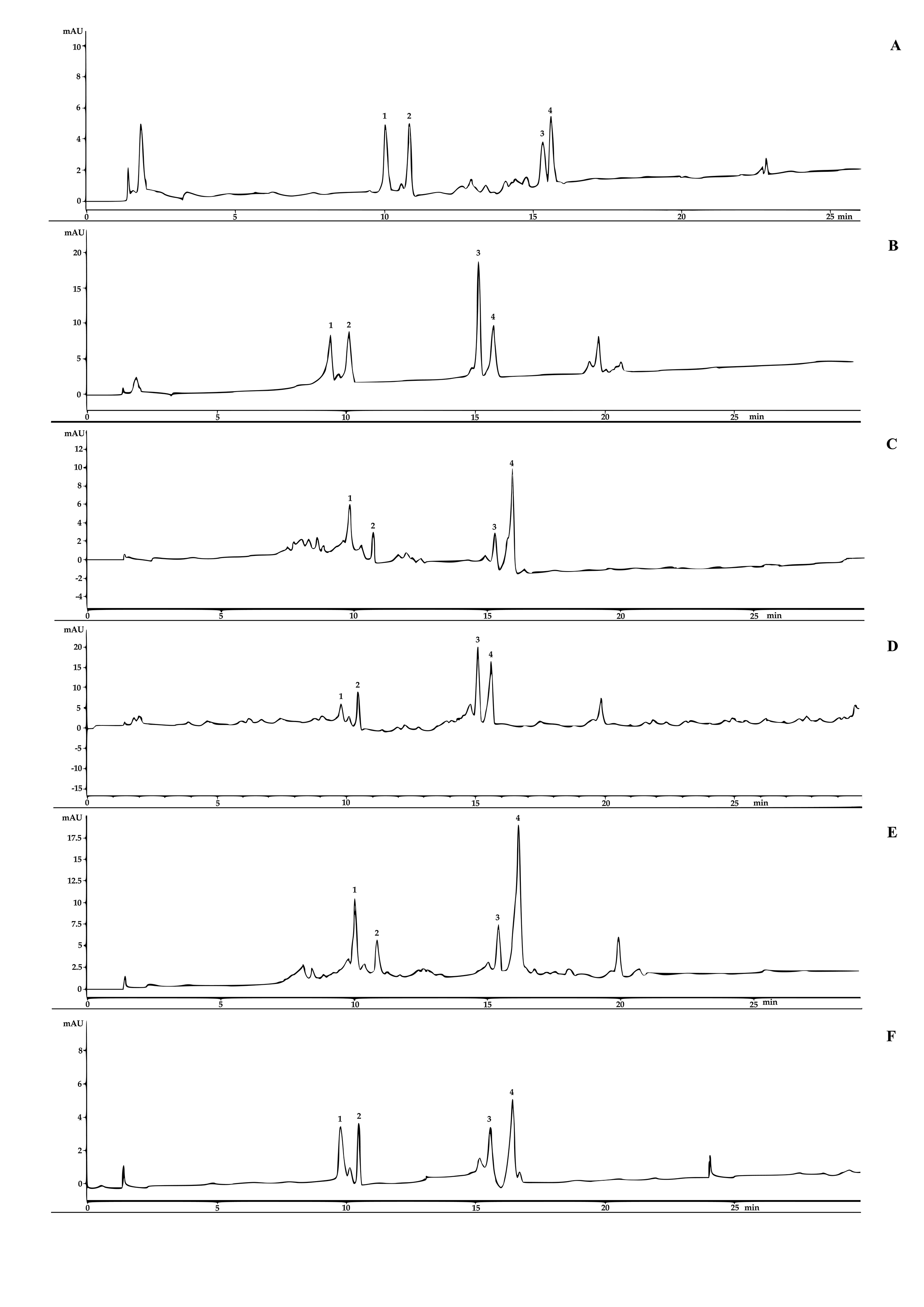

Supplement: Supplementary Figure 1 — Chromatograms of phenolic acids in Genovese basil extract by HPLC at density D2 with separation of caffeic acid (1), ferulic acid (2), chicoric acid (3), and rosmarinic acid (4). (A,B) Aroma 2 at first and second cut. (C,D) Eleonora at first and second cut. (E,F) Italiano Classico at first and second cut. [file Image_1.JPEG]

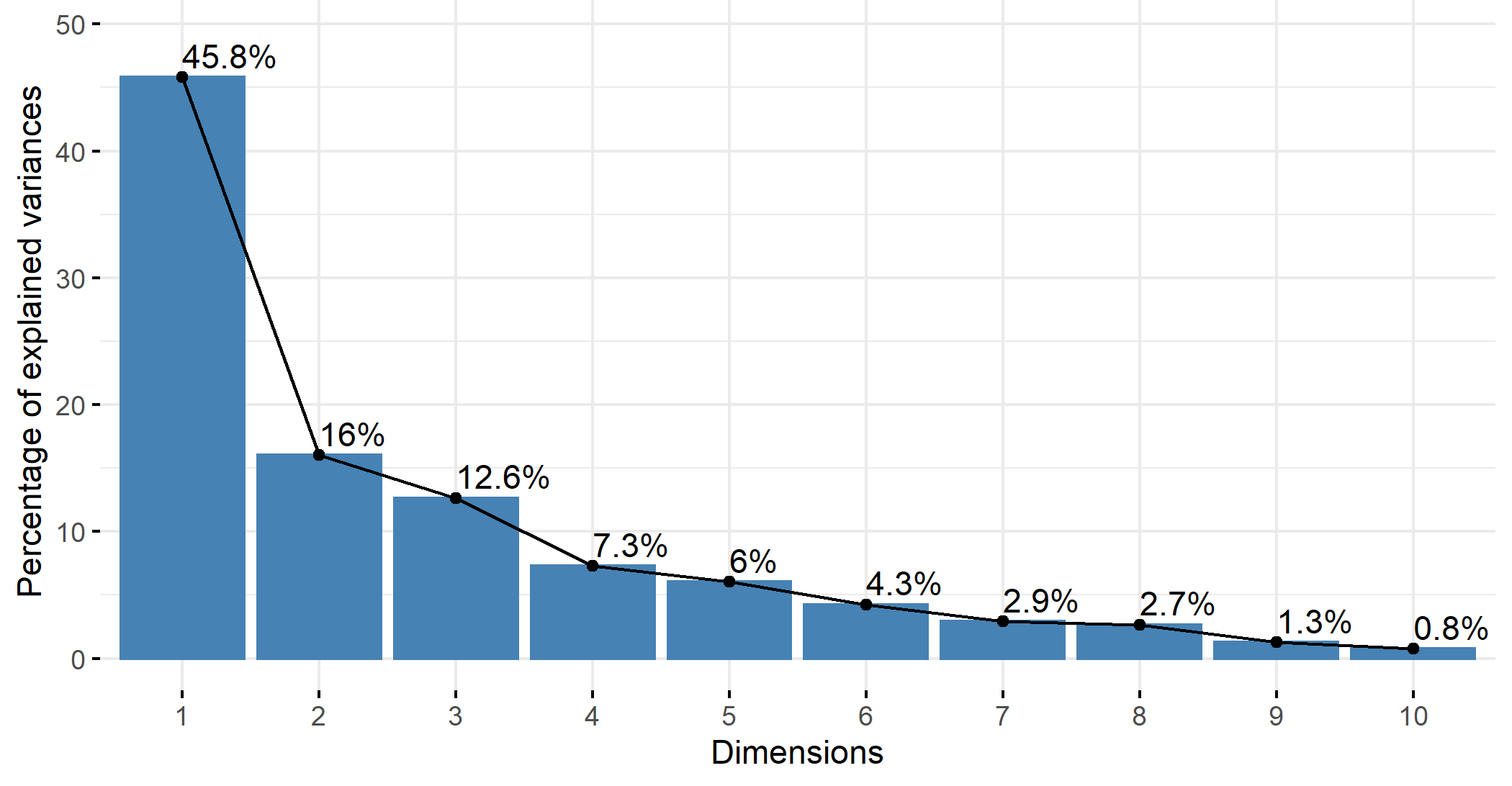

Supplement: Supplementary Figure 2 — Scree plot of the eigenvalues of the principal components. [file Image_2.TIFF]
